# PD-L1/PD-1 and CTLA-4 Expression in Equine Penile Squamous Cell Carcinomas

**DOI:** 10.3390/ani11072121

**Published:** 2021-07-16

**Authors:** Ilaria Porcellato, Samanta Mecocci, Chiara Brachelente, Katia Cappelli, Federico Armando, Alessia Tognoloni, Elisabetta Chiaradia, Valentina Stefanetti, Luca Mechelli, Marco Pepe, Rodolfo Gialletti, Benedetta Passeri, Alessandro Ghelardi, Elisabetta Razzuoli

**Affiliations:** 1 Dipartimento di Medicina Veterinaria, Università degli Studi di Perugia, 06126 Perugia, Italy; ilariaporcellatodvm@gmail.com (I.P.); samanta.mecocci@studenti.unipg.it (S.M.); katia.cappelli@unipg.it (K.C.); alessia.tognoloni@studenti.unipg.it (A.T.); elisabetta.chiaradia@unipg.it (E.C.); valentina.stefanetti@unipg.it (V.S.); luca.mechelli@unipg.it (L.M.); marco.pepe@unipg.it (M.P.); rodolfo.gialletti@unipg.it (R.G.); 2 Centro di Ricerca sul Cavallo Sportivo, Università degli Studi di Perugia, 06126 Perugia, Italy; 3 Dipartimento di Scienze Medico-Veterinarie, Università degli Studi di Parma, 43100 Parma, Italy; federico.armando@unipr.it (F.A.); benedetta.passeri@unipr.it (B.P.); 4 Pathology Institute, University of Veterinary Medicine, 30545 Hannover, Germany; 5 Azienda Usl Toscana Nord-Ovest, UOC Ostetricia e Ginecologia, Ospedale Apuane, 54100 Massa, Italy; alessandro.ghelardi@uslnordovest.toscana.it; 6 IZSLPV, Centro di Referenza di Oncologia Veterinaria e Comparata (CEROVEC), 16129 Genova, Italy; elisabetta.razzuoli@izsto.it

**Keywords:** penile cancer, horse, papillomavirus, PD-L1, PD-1, CTLA-4, carcinoma, squamous cell, animal models, immune checkpoint inhibitors

## Abstract

**Simple Summary:**

In the last few years, the treatment of different types of tumors in humans has benefited from the introduction of new drugs called “immune checkpoint inhibitors” (ICI). These treatments help the patient’s immune response in fighting against cancer, therefore limiting tumor growth and aggressiveness. The possibility to use this therapeutical strategy also in pet animals such as horses has been scarcely explored in veterinary medicine. This study aims at investigating the presence and expression of specific checkpoint molecules such as PD-L1 and CTLA-4 in penile tumors of horses, particularly regarding malignant squamous cell carcinomas. In fact, PD-L1 and CTLA-4 presence is pivotal for an effective response to ICI and a successful therapy. Unfortunately, our results seem to indicate that these molecules are not frequently expressed in equine penile tumors. Therefore, horses with penile tumors may not benefit from the therapeutical use of ICI.

**Abstract:**

In horses, penile squamous cell carcinomas (epSCCs) are among the most common cutaneous neoplastic lesions. These tumors usually arise in benign lesions such as viral plaques and papillomas frequently induced by *Equus caballus* papillomavirus type 2 (EcPV2) infection. In the last decade, the introduction of immune checkpoint inhibitors (ICI) for the treatment of human cancers has demonstrated promising results. Among the most commonly targeted pathways, there is PD-1/PD-L1 and CTLA-4. The aim of this study is to investigate the expression of the PD-1/PD-L1 pathway and CTLA-4 in the tumor microenvironment of epSCCs to assess the feasibility of an immunotherapeutic approach. Twenty equine epithelial tumors were retrospectively selected and submitted to RT-qPCR for PD-1 and PD-L1 genes. After testing antibodies cross-reactivity by western blotting, immunohistochemistry for PD-L1 and CTLA-4 was performed. Results from RT-qPCR demonstrated that 3/20 cases expressed the *PD-L1* gene, whereas the *PD-1* gene was not detected. Immunohistochemical positivity for PD-L1 was found only in one case. CTLA-4-positive cells were observe in all cases but were few (Mdn = 4.8; IQR = 2.3–7.1 cells/HPF). In this study group, PD-1/PD-L1 and CTLA-4 do not appear to be highly expressed and therefore the use of ICI in epSCCs may not have promising rates of response.

## 1. Introduction

Equine penile squamous cell carcinomas (epSCCs) are the most common tumors of the external genitalia with a reported incidence of 49–82.5% [1]. These tumors commonly arise on viral plaques or papillomatous lesions of older horses (17.4–19.5 years), can cause discomfort and the possibility of a local recurrence after surgical excision or distant metastases that can lead the animal to death [2,3]. Most of these tumors are now recognized to be caused by a specific type of papillomavirus (PV) called *Equus caballus* papillomavirus type 2 (EcPV2) [4].

Similarly, in humans, penile SCCs can be caused by high-risk HPV, even though the percentage of PV-positive tumors seems to be lower than in horses [5]. Different studies provided evidence of similarities among human and equine penile squamous cell carcinomas, suggesting that the horse could represent a good preclinical model for the human counterpart [6,7,8].

During the last few years, cancer immunotherapy has demonstrated promising results with different types of tumors [9,10,11,12]. In particular, with the introduction of immune checkpoint inhibitor (ICI)-targeted immunotherapy, the immunotolerant tumor microenvironment can be overcome by reactivating T cells, enhancing anti-tumor immunity, and eliminating tumor cells more effectively [13].

In the last decade, programmed death protein-1/programmed death-ligand-1 (PD-1/PD-L1) and cytotoxic T lymphocyte antigen 4 (CTLA-4) have become two of the most important pathways targeted by ICI and are involved in the induction and maintenance of immunotolerance within the tumor microenvironment [14].

PD-1 (also known as CD279) is a membrane co-inhibitor receptor expressed on activated T cells, natural killer cells, B lymphocytes, regulatory T cells (Tregs), antigen presenting cells (APCs), monocytes, vascular endothelial cells, and mesenchymal stem cells [14,15]. This molecule plays a pivotal role in regulating T cell immunity by preventing their overactivation and maintaining their exhaustion in chronic inflammatory processes but can also be exploited by tumor cells to evade immune responses [16].

PD-L1 (also known as CD274 or B7-H1) is a member of the B7 family and is expressed by APCs (both macrophages and dendritic cells), and are also activated and exhausted T and B lymphocytes and Tregs. Moreover, PD-L1 expression has been observed in cardiac endothelium and placenta and pancreatic islets, where its role is likely in the maintenance of immunological tolerance [17]. Its expression has been frequently observed in human and animal cancers [18,19,20,21,22].

In human penile SCCs (peSCCs), the role of PD-1/PD-L1 has been explored by some studies that have emphasized that the expression of PD-L1 was associated with unfavorable disease-specific survival and that tumors with a diffuse PD-L1 tumor cell expression had a worse prognosis when compared to those with a marginal or negative PD-L1 expression. Moreover, it appears that PD-L1 was more expressed in HPV-tumors rather than in HPV+ ones [23,24,25]. On the contrary, HPV-positive peSCC exhibited a higher density of intratumoral PD-1 than the HPV-negative tumors [25].

CTLA-4 (also known as CD152) is a protein receptor expressed on activated T cells and Tregs. It binds to CD80 and CD86, expressed by antigen presenting cells and leading to a downregulation of T cell activation [26,27,28]. Together with PD-1, CTLA-4 is one of the main targets of ICI, having demonstrated to be pivotal in the reversion on T cell tolerance in tumor microenvironment and in the treatment of autoimmune diseases [29].

A recent study by Schoenfeld and colleagues on neoadjuvant immunotherapy combining PD-1 and CTLA-4 ICI and given prior to surgery on human patients with oral SCCs demonstrated promising rates of response, supporting further studies using this approach [30].

To the best of the authors’ knowledge, PD-1/PDL1 pathway and CTLA-4 expression have never been investigated in equine penile SCCs. The aim of the present study is to investigate the expression of these molecules in the tumor microenvironment of epSCCs to assess the feasibility of a future immunotherapeutic approach in these tumors.

## 2. Materials and Methods

### 2.1. Case Selection

Cases for this study were retrospectively selected (2005–2019) from the formalin-fixed and paraffin-embedded (FFPE) archives of the Department of Veterinary Medicine of the University of Perugia and of the Department of Veterinary Science of the University of Parma with the following inclusion criteria:

- histological diagnosis of equine papilloma, carcinoma in situ, and squamous cell carcinoma assessed by experienced pathologists (IP, CB, BP) as per recently suggested diagnostic criteria [1,3];
- confirmed penile localization of the lesions;
- and availability of >0.5 cm^2^ of FFPE tumor tissue evaluated on section.

### 2.2. DNA Extraction and EcPV2 Detection

Two to four 5 μm-thick sections were cut from selected FFPE blocks (one for each case) and subjected to DNA extraction to evaluate for EcPV2 presence. The AllPrep DNA FFPE Kit (Qiagen, Venlo, The Netherlands) was used as per the manufacturer’s instructions. Previously described primers for EcPV2-*L1* DNA together with specific probes were used for virus detection and the Beta-2-Microglobulin (*B2M*) gene was amplified to assess DNA integrity. Previously reported primer sequences [7,8] are detailed in Table 1.

Real-time PCR was performed in a CFX96™ Real-Time System using the Horse-Power™ Taq DNA Polymerase MasterMix (Canvax Biotech, Cordoba, Spain), 200 nM of the probe, 100 nM of each primer, and 100 ng of DNA. A threshold cycle of 38 was set as the cut-off for virus positivity and samples were considered negative with Cq > 38.

### 2.3. RNA Extraction and Real-Time PCR

Total RNA extraction of each FFPE sample was performed from 5 or 6 sections (5 µm) using the RecoverAll™ Total Nucleic Acid Isolation Kit for FFPE (Invitrogen, ThermoFisher Scientific, Waltham, MA, USA) according to manufacturer’s instructions and evaluated for RNA concentrations through a NanoDrop 2000 (Thermo Fisher Scientific, Waltham, MA, USA) spectrophotometer.

The reverse transcription (RT) step was performed using the SuperScript™ IV VILO™ Master Mix (Invitrogen, ThermoFisher Scientific, Waltham, MA, USA), adding 250 ng of RNA and utilizing 5 μL of 1:5 diluted cDNA.

*L1* and *E6* EcPV2 genes were tested for their expression using specific primer sets and probed (Table 1) at the concentration of 200 nM, and 1 μM for each primer combination was added to the 25 μL PCR mixture at the final concentration of 1× master mix (iTaq Universal ProbsSupermix, Bio-Rad, Irvine, CA, USA) with the following thermal profile: 95 °C for 10′, then 39 cycles of 95 °C for 15′′ and 60 °C for 60′′ in a CFX96™ Real-Time System. RNA was used as the control to exclude possible contaminations by EcPV2 genomic DNA.

*PD-1* and *PD-L1* were selected to test their related gene expression. Primers for the PD-1 gene *(PDCD1*) and *PD-L1* gene (*CD274*) were designed through Primer3web tool v. 4.1.0 [31] and reported in Table 1. *B2M* gene expression was used to normalize the host gene expression [32] utilizing primers previously reported (Table 1). The amplification was conducted using the SsoFast EvaGreen Supermix (BioRad, Hercules, CA, USA) in a CFX96 Real-Time System with 5 μL of 1:5 diluted cDNA. Each sample was tested in triplicates and fluorescence data were collected at the end of the second step of each cycle. A threshold cycle of 38 was set as the cut-off for positivity and samples with Cq > 38 were considered negative. All PCR products including the positive control were subjected to direct sequencing.

### 2.4. Western Blotting

Before immunohistochemical analysis, anti-human PD-L1 (GXGTX31308, GeneTex, Irvine, CA, USA) and CTLA-4 (clone F-8, Santa Cruz Biotechnology, Dallas, TX, USA) antibodies were validated for the detection of the equine proteins in the IHC by western blotting (WB) [33].

A fresh lymph node and liver were obtained during necropsy from healthy horses. The placenta, obtained from a 12-year-old Thoroughbred mare, was collected within 2 h of expulsion following an eutocic delivery at the end of a physiologically normal gestation length. All the samples were stored at −80 °C until further investigation.

Afterwards, samples were homogenized in a lysis buffer (Cell Signaling) and the proteins obtained after centrifugation at 4 °C for 13,000× *g* for 15 min were quantified using the Bradford assay. 25 µg of total proteins of each tissue were separated by polyacrylamide gel electrophoresis (SDS-PAGE) 11% T and then transferred on PVDF membranes. Specific protein bands were detected incubating membranes with the rabbit anti-human PD-L1 (1:1000) or mouse CTLA-4 antibody (1:1000) overnight at 4 °C and then at room temperature for 90 min with the appropriate IgG polyclonal antibodies (1:5000) (Santa Cruz Biotechnology).

The immuno-complexes were evidenced using the Clarity Western ECL Substrate (Bio-Rad, Hercules, CA, USA) and by exposing X-ray films. Images were acquired using a GS-800 imaging systems scanner (Bio-Rad, Hercules, CA, USA).

### 2.5. Immunohistochemistry

Immunohistochemical labeling was performed on 5 µm serial sections mounted on poly-L-lysine coated slides. Briefly, after deparaffination, antigen retrieval was performed in a microwave by immersion in a pre-heated Tris-EDTA (pH 9.0). Then, slides were washed with PBS and incubated in 3% H_2_O_2_ for 10 min. After a protein blocking step, the incubation with rabbit polyclonal anti-PD-L1 and mouse monoclonal anti-CTLA-4 [34] antibodies was performed. After secondary biotinylated antibody and streptavidin incubation, immunolabeling was revealed with AEC.

Positive cells were counted on 5 randomly selected high-power fields, avoiding areas near ulceration or necrosis.

Equine placenta and equine lymph node were used as controls for PD-L1 and CTLA-4, respectively. Negative controls were conducted by omitting the primary antibody and incubating the slides with PBS.

## 3. Results

### 3.1. Case Selection and Histology

Twenty cases of equine penile epithelial tumors matching the inclusion criteria were retrospectively selected. Horses had between 2 and 34 years of age at the moment of the diagnosis (mean 18.7 years). In six cases the lesion was on the glans and in six cases on the prepuce, whereas in the remaining cases the exact penile site of origin was not specified.

Results from histopathological revision of cases confirmed the original diagnosis of epithelial tumor and in particular one case was diagnosed as a papilloma; 2/20 were confirmed to be carcinomas in situ, whereas the remaining cases (17/20) were classified as invasive squamous cell carcinomas. The cases reported in this study are overlapping with the ones reported in previous studies [7,8].

### 3.2. DNA Extraction and EcPV2 Detection

The *B2M* gene was amplified in all of the samples and were therefore considered to be suitable for the investigation of the viral gene L1 and E6. Eighteen (18) out of twenty (90%) cases were positive for both the EcPV2-*L1* and EcPV2-*E6* virus DNA, whereas in two cases both genes tested negative. The real-time PCR of EcPV2-*L1* and EcPV2-*E6* transcripts demonstrated a positivity of 60% (12/20) and 55% (11/20), respectively (Table 2).

### 3.3. PD-1 and PD-L1 Gene Expression

Results obtained from the real-time PCR demonstrated that 3 out of 20 tested cases expressed *CD274* (PD-L1 gene), while for *PDCD1* (PD-1 gene) no positive sample was detected with a threshold lower than 38 Cq (Table 2). The amplicons resulted by direct sequences were submitted to a Basic Local Alignment Search Tool for analysis (https://blast.ncbi.nlm.nih.gov/Blast.cgi, accessed on 12 July 2021) to verify specific amplification and after alignments the sequences matched perfectly with the Equus caballus *CD274* gene.

### 3.4. Western Blotting

**Figure 1:**
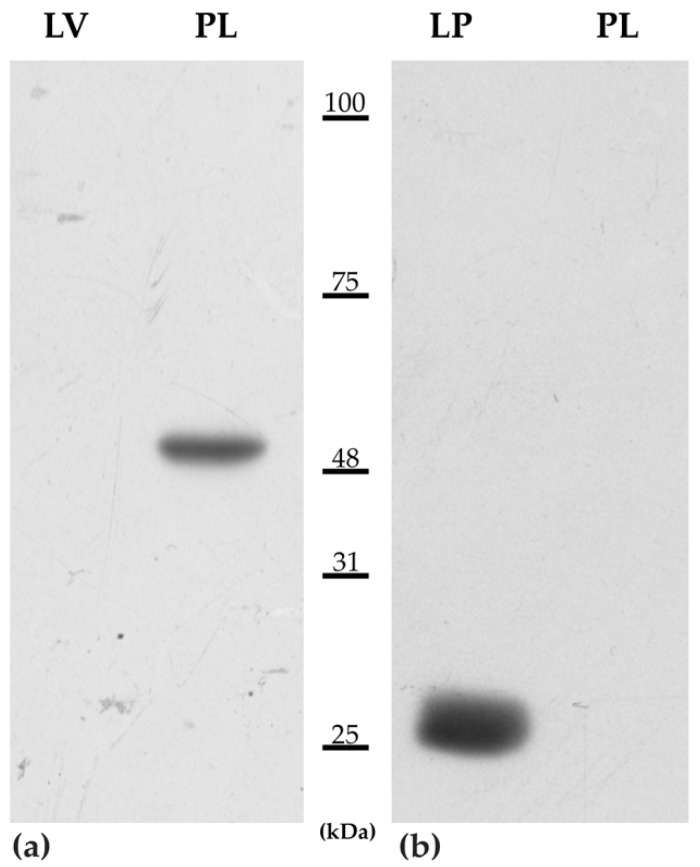
Representative immunoblotting images obtained separating proteins extracted from the equine liver (LV), placenta (PL), and lymph node (LP) on SDS-PAGE 11% T, and detected the immunoreactive bands using the rabbit antibody PD-L1 (**a**) and mouse anti-human CTLA-4 (**b**).

The WB analysis (Figure 1) indicated that the anti-human PD-L1 and anti-CTLA-4 antibodies tested cross-reacted with the corresponding equine proteins. A single band corresponding to approximately the expected molecular weight of the equine PD-L1 (42.4−50.3 kDa) [35] was detected in the placenta, whereas no positive signal was observed in the liver lane. A single band of approximately 25 kDa corresponding to the expected molecular weight of CTLA-4 (https://www.uniprot.org/uniprot/F6WWE3, accessed on 23 April 2021) was detected in the lymph node but was negative in the placenta lane. Furthermore, no extraneous bands were detected in any of the samples investigated.

### 3.5. Immunohistochemistry

**Figure 2:**
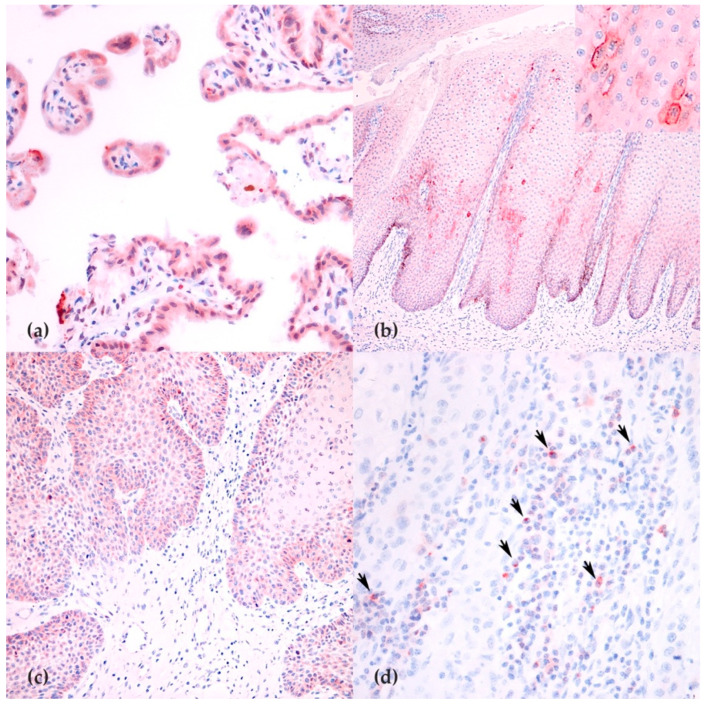
PD-L1 and CTLA-4 expression in equine tissues: (**a**) placental expression of PD-L1 was observed as a cytoplasmic positivity (400×); (**b**) expression of PD-L1 in hyperplastic mucosa near a SCC (100×); (**c**) diffuse expression of PD-L1 in the invasive front of a SCCs (200×); and (**d**) intratumoral lymphocytes expressing CTLA-4 (400×) (arrows).

The expression of PD-L1 was observed on chorionic placental villi (Figure 2a) with a reaction pattern similar to what previously reported in horses and humans [36,37]. Immunohistochemical analysis was performed on 17/20 cases, as in three cases the tissue was considered not sufficient after serial recuts.

Positivity for PD-L1 was observed only in one sample (case 18; Figure 2c) that revealed on RT-qPCR the highest expression for this gene. Moreover, PD-L1 was occasionally observed on hyperplastic epithelium, far from the neoplastic cells (Figure 2b).

CTLA-4 expression was observed as a membranous granular reactivity on small lymphocytes within the equine lymph node. The presence of scattered CTLA-4-positive cells was assessed in all 17/20 tested equine tumors (Figure 2d). The median number of positive cells/HPF was 4.8 (IQR 2.3–7.1).

## 4. Discussion

Penile tumors are quite common in older horses, particularly squamous cell carcinomas, that can cause discomfort to the animal or even more serious complications, such as local recurrence after surgery or local and distant metastasis [1,3]. Therapy can vary from topical treatment to en bloc penile resection; ideally, the treatment should eliminate the tumor without impairing the functions of the external genitalia [1].

The introduction of immune therapy in human medicine has been a revolution in cancer therapy during the last decade, bearing unprecedented successes. In particular, immune checkpoint inhibitors (ICI) have demonstrated good results in several tumors both when used in monotherapy and in combination. Still, not all cancer types respond well to ICI and therefore further studies are recommended [38].

The aim of this study was to investigate the expression of two of the most important targets of the ICI: PD-1/PD-L1 pathway and CTLA-4 in equine penile squamous cell carcinomas (epSCCs) to assess the feasibility of a future immunotherapeutic approach.

In our study, the expression of *PDCD1* (PD-1 gene) was not observed setting the threshold of 38 cycles on RT-qPCR; indeed, in the majority of samples the signal was detected at a higher cycle number. However, it cannot be ignored that this result could be due to the poor quality of RNA extracted from FFPE.

PD-1 is known to be a T-cell checkpoint that inhibits the activation and activity of effector T cells. In epSCCs, the low expression of this molecule might indicate the presence of an active immune environment compatible with the observation of a vivacious tumor immune environment as previously described [7]. This finding seems to be in contrast with what was reported in humans, where PD-1 seems to be upregulated within the microenvironment of HPV+ tumors [25]. The results of our study may be caused by partial degradation of RNA that is common in FFPE samples. Therefore, further data on fresh tissues collected in prospective studies would be necessary to confirm this preliminary finding.

In addition, the expression of *CD274* (PD-L1 gene) was limited in epSCCs as this gene was expressed only in 3/20 cases. One of these three cases confirmed the gene expression at the protein level and exhibited a multifocal to coalescing immunolabelling of tumor cells on immunohistochemistry. Interestingly, this case was also negative for EcPV2, similar to what is commonly reported in human medicine. In fact, different studies on the human counterpart have demonstrated that the expression of PD-L1 is mostly observed in HPV-negative tumors rather than in those induced by papillomavirus [24]. Moreover, the patients with PD-L1 tumor expression had a worse prognosis than those with marginal or negative PD-L1 expression [24,25]. Most of the tumors of our study group were positive for EcPV2 and were therefore considered EcPV-induced. As these tumors were mostly negative for PD-L1, the results of this study support epSCC as a good model for the human HPV-induced penile squamous cell carcinomas [6,7], whereas more data are required to confirm that also HPV-negative tumors are similar to the human counterpart.

The presence of few CTLA-4 positive cells within the tumor microenvironment in the context of a negativity of tumor cells seems to indicate that this pathway is not particularly involved in the immunoescape of epSCCs. Still, further studies on CTLA-4 expression in veterinary neoplastic diseases are necessary as currently available data are still few [34,39,40]. To the best of the authors’ knowledge, this was the first time CTLA-4 was investigated in horses.

This study has some limitations, in particular due to the use of retrospectively selected formalin-fixed and paraffin-embedded samples. Fresh samples would allow more robust results with RT-qPCR by limiting nucleic acid degradation. Additionally, the collection of a complete follow-up that in the present study is missing would contribute information on the tumor’s biological behavior and would provide data for the evaluation of prognostic markers. Moreover, a higher number of papillomas and CIS should be included in a future study to evaluate possible differences with SCCs.

Further prospective studies with a larger case group would be beneficial both for the improvement of the therapy of epSCCs and for gathering information on the human counterpart, using the horse as a spontaneous model of the disease.

## 5. Conclusions

In this study group, PD-1/PD-L1 and CTLA-4 do not appear to be highly expressed and therefore the use of ICI in epSCCs may not have promising rates of response.

The low rate of expression of PD-L1 in equine penile SCCs and epithelial tumors seems to mirror human HPV-induced penile cancers phenotypical characteristics, therefore representing a possible good comparative model for this subgroup of tumors.

## Figures and Tables

**Table 1:** Primer pairs and probe details.

Gene	Primer Pairs Sequences	Amplicon Length	Accession and References
EcPV2-*L1*	F-5′- TTGTCCAGGAGAGGGGTTAG-3′	81	NC_012123 [7]
	R-5′- TGCCTTCCTTTTCTTGGTGG-3′		
pEcPV2-*L1*	FAM-CGTCCAGCACCTTCGACCACCA-TAMRA		
EcPV2-*E6*	F-5′-CGTTGGCCTTCTTTGCATCT-3′	81	NC_012123 [8]
	R-5′-AGGTTCAGGTCTGCTGTGTT-3′		
p-EcPV2-*E6*	FAM- CCGTGTGGCTATGCTGATGACATTTGG-TAMRA		
Ec-B2M DNA detection	F-5′-CTGATGTTCTCCAGGTGTTCC-3′	114	NM_001082502 [7]
	R-5′-TCAATCTCAGGCGGATGGAA-3′		
p-*B2M*	FAM-ACTCACGTCACCCAGCAGAGA-TAMRA		
Ec-*PDCD1* (PD1)	F-5′-GCCTGTGTCCTGACCACC-3′	150	XM_023642815
	R-5′-CTCCGGGGTCTTCTCTCG-3′		
Ec-*CD274* (PDL1)	F-5′-GTGCTGACTACAAGCGGATT-3′	120	XM_001492842
	R-5′-GGTAACCCTCAGCCTGACAT-3′		
Ec-*B2M cDNA*	F-5′-TCCTGCTCGGGCTACTCTC-3′	83	NM_001082502 [7]
	R-5′-TGCTGGGTGACGTGAGTAAA-3′		

**Table 2:** Histological diagnosis: SCC: squamous cell carcinoma; CIS: carcinoma in situ; P: papilloma. Real-time PCR data for *B2M* are expressed as + (amplified). Viral DNA detection and gene expression were given indicating the mean Cq at which the positivity for *L1* and *E6* were detected: + (32–37 Cq), ++ (26–31 Cq), +++ (20–25 Cq), and ++++ (14–19 Cq), and ND (not detected). For equine genes, the mean of normalized expression ± 1 standard error of three replicates is reported for *PDCD1* and *CD274*; samples with a mean Cq > 38 were considered negative.

Case ID	Histological Diagnosis	DNA			cDNA		Normalized Expression	
		*B2M*	*L1*	*E6*	*L1*	*E6*	*PDCD1*	*CD274*
1	SCC	+	+++	++	+	++	Negative	ND
2	SCC	+	+	+	ND	ND	ND	5.3 ± 7.3
3	CIS	+	+++	++	+	+	ND	Negative
4	SCC	+	++++	+++	+	+	ND	ND
6	P	+	+	+	ND	ND	Negative	ND
7	SCC	+	+	+	ND	ND	Negative	ND
8	SCC	+	++++	+++	+	+	Negative	ND
9	SCC	+	++++	+++	+	+	Negative	Negative
10	SCC	+	++++	+++	+	+	Negative	ND
11	SCC	+	+	+	ND	ND	Negative	ND
12	SCC	+	++++	+++	+	+	Negative	ND
13	SCC	+	+	+	ND	ND	Negative	ND
14	SCC	+	++	+	+	ND	ND	ND
15	SCC	+	++++	+++++	++	+	Negative	ND
17	SCC	+	++++	+++	+	+	Negative	ND
18	SCC	+	++	++	ND	ND	Negative	0.6 ± 0.7
20	SCC	+	ND	ND	ND	ND	Negative	Negative
21	CIS	+	++++	+++	++	++	ND	9.3 ± 8.4
23	SCC	+	+++	++++	+	+	Negative	ND
24	SCC	+	ND	ND	ND	ND	Negative	ND

## Data Availability

Not applicable.
